# Prioritization of pig farm biosecurity for control of *Salmonella* and hepatitis E virus infections: results of a European expert opinion elicitation

**DOI:** 10.1186/s40813-023-00306-0

**Published:** 2023-03-06

**Authors:** Erika Galipó, Veit Zoche-Golob, Elena Lucia Sassu, Christopher Prigge, Marie Sjölund, Tijs Tobias, Artur Rzeżutka, Richard Piers Smith, Elke Burow

**Affiliations:** 1 Animal and Plant Health Agency, Woodham Lane, New Haw, Addlestone, KT15 3NB UK; 2 Department of Biological Safety, German Federal Institute for Risk Assessment (BfR), Max-Dohrn-Str. 8-10, 10589 Berlin, Germany; 3 Division for Animal Health, Austrian Agency for Health and Food Safety, Robert-Koch-Gasse 17, 2340 Mödling, Austria; 4 Unit of Veterinary Public Health and Epidemiology, Institute of Food Safety, Food Technology and Veterinary Public Health, University of Veterinary Medicine, Veterinärplatz 1, 1210 Vienna, Austria; 5 Department of Animal Health and Antimicrobial Strategies, National Veterinary Institute, 751 89 Uppsala, Sweden; 6 Department of Clinical Sciences, Swedish University of Agricultural Sciences, P.O. Box 7054, 750 07 Uppsala, Sweden; 7 Department of Population Health Sciences, Faculty of Veterinary Medicine, Utrecht University, Yalelaan 7, 3584 CL Utrecht, The Netherlands; 8 Department of Food and Environmental Virology, National Veterinary Research Institute, Al. Partyzantów 57, 24-100 Puławy, Poland

**Keywords:** *Salmonella*, HEV, Biosecurity measure, Pig farming, Expert opinion elicitation (EOE), Foodborne diseases

## Abstract

**Background:**

In the literature, there is absent or weak evidence on the effectiveness of biosecurity measures to the control of *Salmonella* spp. and hepatitis E virus (HEV) on pig farms. Therefore, the present study aimed to collect, weigh, and compare opinions from experts on the relevance of several biosecurity measures. An online questionnaire was submitted to selected experts, from multiple European countries, knowledgeable on either HEV or *Salmonella* spp.*,* in either indoor or outdoor pig farming systems (*settings*). The experts ranked the relevance of eight biosecurity categories with regards to effectiveness in reducing the two pathogens separately, by assigning a score from a total of 80, and within each biosecurity category they scored the relevance of specific biosecurity measures (scale 1–5). Agreement among experts was analysed across pathogens and across *settings*.

**Results:**

After filtering for completeness and expertise, 46 responses were analysed, with 52% of the experts identified as researchers/scientists, whereas the remaining 48% consisted of non-researchers, veterinary practitioners and advisors, governmental staff, and consultant/industrial experts. The experts self-declared their level of knowledge but neither Multidimensional Scaling nor k-means cluster analyses produced evidence of an association between expertise and the biosecurity answers, and so all experts’ responses were analysed together without weighting or adaptation. Overall, the top-ranked biosecurity categories were *pig mixing*; *cleaning and disinfection*; *feed, water and bedding*; and *purchase of pigs or semen*, while the lowest ranked categories were *transport*, *equipment*, *animals* (other than pigs and including wildlife) and *humans*. *Cleaning and disinfection* was ranked highest for both pathogens in the indoor setting, whereas *pig mixing* was highest for outdoor *settings*. Several (94/222, 42.3%) measures across all four *settings* were considered highly relevant. Measures with high disagreement between the respondents were uncommon (21/222, 9.6%), but more frequent for HEV compared to *Salmonella* spp.

**Conclusions:**

The implementation of measures from multiple biosecurity categories was considered important to control *Salmonella* spp. and HEV on farms, and pig mixing activities, as well as cleaning and disinfection practices, were perceived as consistently more important than others. Similarities and differences in the prioritised biosecurity measures were identified between indoor and outdoor systems and pathogens. The study identified the need for further research especially for control of HEV and for biosecurity in outdoor farming.

**Supplementary Information:**

The online version contains supplementary material available at 10.1186/s40813-023-00306-0.

## Introduction

Although slaughter procedures can reduce contamination and the risk of foodborne diseases, pre-harvest food safety is deemed ‘important’ to control the risk to people [1]. Amongst other pathogens, *Salmonella* spp*.* and hepatitis E virus (HEV) are zoonotic pathogens which stand out in the pig production for their impact on human health and difficulty to control on pig farms. *Salmonella* spp*.* infections in humans are mostly characterized by gastrointestinal disorders but rarely cause clinical signs in pigs. About 10–20% of the human infections with *Salmonella* spp. in EU may be attributable to the pig reservoir [2]. HEV infection is typically subclinical in pigs [3] as well as in humans, but in humans it can lead to an acute or acute-on-chronic hepatitis in immunocompromised individuals [4]. In Europe, pigs and wild boars are the main reservoirs of HEV genotypes 3 and 4 which are zoonotic and, although the source of human infection is mostly unknown, the foodborne route is considered important for HEV-3 and -4 infections [5].

In the past 20 years, it has become apparent how biosecurity, defined as the holistic approach in managing biological risk through a set of policy frameworks and preventive measures [6], can help in reducing the burden of foodborne pathogens in pig farming [7, 8]. Nonetheless, for some pathogens scientific evidence on the effect of individual biosecurity measures under field conditions is scarce or lacking quantification. Biosecurity measures relevant to control HEV occurrence on pig farms are not yet comprehensively studied, but evidence from other pathogens may be relevant as HEV may share a common infection route [9, 10]. For *Salmonella* spp*.,* although pathogen specific practices may be known and studied [11–18], effectiveness of biosecurity measures is also affected by farming type and prevalence. However, in some cases information is lacking for newer, and more welfare friendly, housing systems, for instance with outdoor housing. Despite the general understanding that biosecurity protocols are highly important tools to reduce the load of *Salmonella* spp*.* and HEV along the food chain, agreement on effectiveness of biosecurity protocols for pig farming is lacking, highlighting the need of a comprehensive protocol for specific conditions (production stage, production system, and prevalence status).

When quantitative information is lacking or there is great variation in the scientific outputs, expert opinion elicitation (EOE) has been undertaken on multiple occasions in the scientific domain, and it is deemed valid and valuable in the absence of systematic evidence [19, 20]. There are several accepted formats to elicit the expert opinion (Likert scale, ranking, rating scales, conjoint analysis, etc.) and they have been used in the veterinary and human science to close the information gap on risk factors for disease freedom assessment, risk assessment and to inform disease control strategies in different livestock industry and for multiple pathogens [19–25]. The present study was developed within the BIOPIGEE project (https://onehealthejp.eu/jrp-biopigee/) and its objectives were to a) collect, weigh, and compare opinions from experts from multiple European countries on the relevance of different biosecurity measures in the control of *Salmonella* spp*.* and HEV, within the indoor and outdoor pig farming systems; b) provide a ranked list that helps to prioritize biosecurity measures most effective in tackling the on-farm circulation of the two pathogens under investigation, and thus support the reduction of the risk of foodborne exposure to people.

## Results

The online questionnaire was answered by 62 of the 83 invited experts. However, 4 respondents did not answer any of the questions relative to a *setting*, and another 11 respondents completed less than 70% of the questions per selected section. These 15 responses were excluded. An additional response was excluded where only parts were completed for which the expert indicated her-/himself as being “not knowledgeable at all”. After filtering for completeness and expertise, answers from 46 experts were kept.

### Experts

About half of the experts identified themselves as *researchers/scientists* (24/46, 52%). The remaining half consisted of *non-researchers* (22/46, 48%), and that was further broken down into *veterinary practitioners and advisors* (11/46, 24%), *governmental staff* (7/46, 15%), and *consultant/industrial experts* (4/46, 9%). The experts were mainly from Germany (11/46, 24%), Poland (7/46, 15%), UK (6/46, 13%), Austria (5/46, 11%), Italy (5/46, 11%) and others (12/46, 26%) (Additional file 1: Table S1).

### Self-assessed knowledge and selected questionnaire parts

**Fig. 1:**
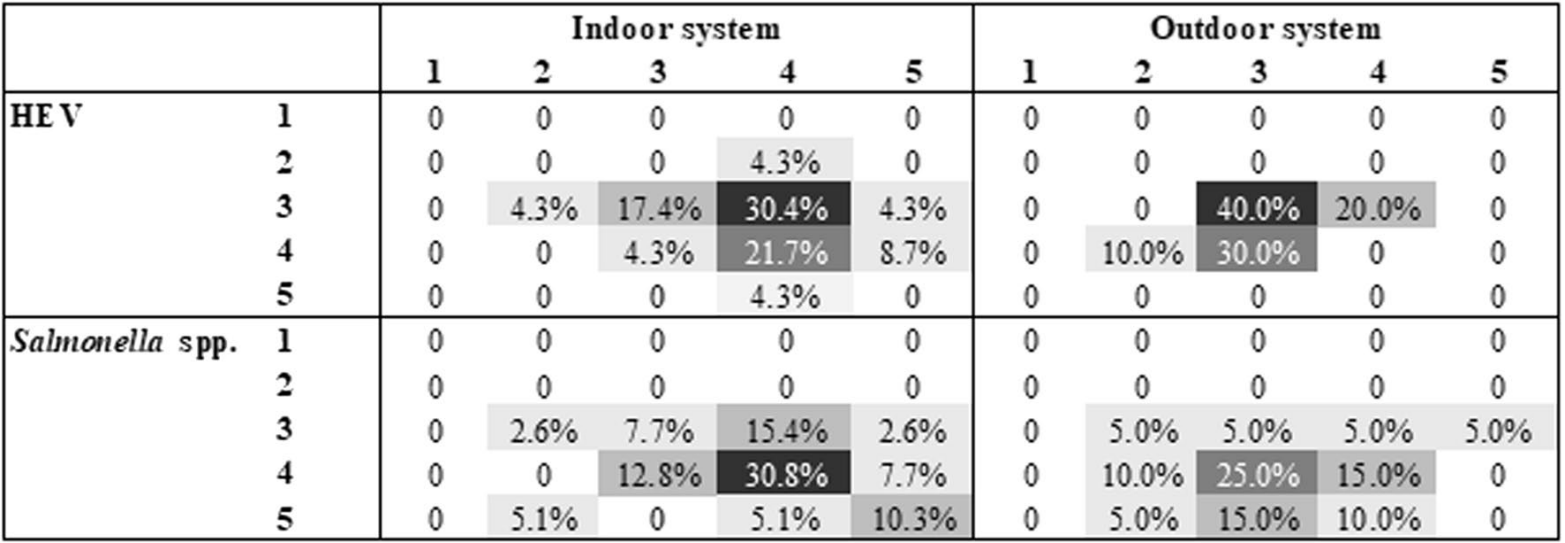
Distribution of participants subset who answered questions relevant for each *setting* in the self-assessed knowledge ranks. The total number of respondents varies: *Salmonella* spp*.* × indoor (n = 39), *Salmonella* spp*.* × outdoor (n = 20), HEV × indoor (n = 23), HEV × outdoor (n = 10). Legend: 1 = not knowledgeable at all, 2 = slightly knowledgeable, 3 = moderately knowledgeable, 4 = very knowledgeable, 5 = extremely knowledgeable. Colour legend, cut-offs were defined based on the percent of total responses per *setting*, at: 1.0–10.0%; 10.1–20.0%; 20.1–30.0%; 30.1–40.0% (from lighter to darker grey shade)

More respondents completed the questionnaire for the setting of SAL-IN (39/46, 84.8%), followed by HEV-IN (23/46, 50.0%), SAL-OUT (20/46, 43.5%) and HEV-OUT (10/46, 21.7%). All the possible combinations of selected *settings* are displayed in Additional file 1: Table S2. In terms of self-assessed knowledge, the most frequently selected combinations were very knowledgeable in SAL-IN (12/39, 30.8%), moderately knowledgeable in SAL-OUT*.* (9/20, 25.0%), very knowledgeable HEV-IN (7/23, 30.4%), and moderately knowledgeable in HEV-OUT (4/10, 40.0%), as shown in Fig. 1. A combination of self-assessed knowledge in both pathogen and system for all the participants to the EOE (n = 46) is provided in Additional file 1: Figure S1; responses relative to indoor husbandry systems, outdoor husbandry systems, *Salmonella* spp*.*, HEV, and on-farm biosecurity in isolation are provided in Additional file 1: Table S3.

### Relevance of biosecurity categories and individual biosecurity measures

**Fig. 2:**
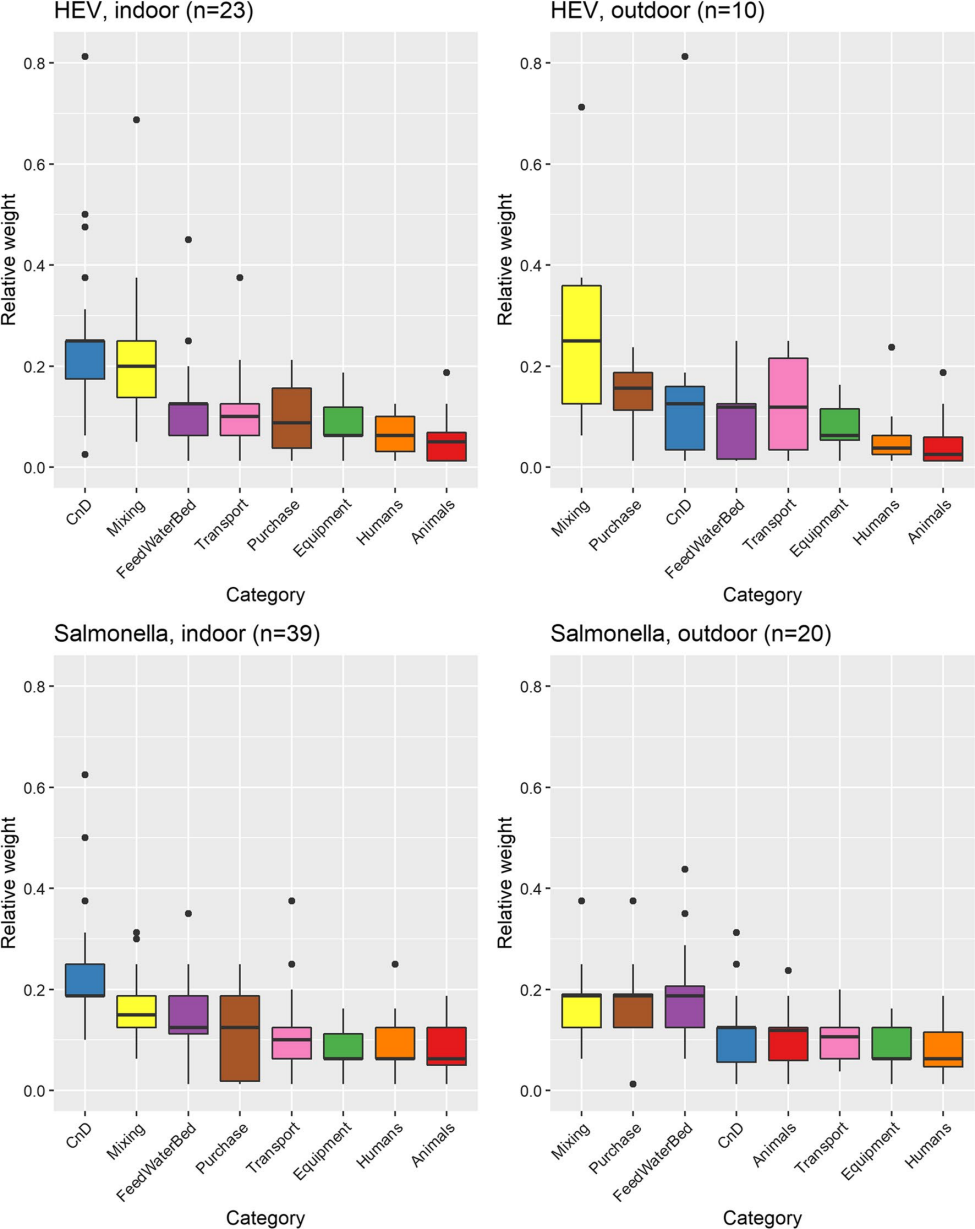
Box plots representing the relative weights of the biosecurity measures (ranked by median relevance from largest to smallest, and interquartile range, from smallest to largest). Legend: CnD indicates cleaning and disinfection; Mixing indicates mixing pigs, Feed, water and bed indicates feed. water supply, and bedding; Transport indicates transport to/from farm; Purchase indicates purchase of pigs or semen; Equipment indicates material and equipment; Humans indicates farm workers and visitors; Animals indicates other animal species on farm, including wildlife

Neither MDS nor k-means cluster analyses resulted in evidence of an association between the self-assessed level of expertise and the answers to the questions about biosecurity, and so all experts’ responses were analysed together without weighting or adaptation. With regards to SAL-IN*,* the biosecurity category ranked highest was *cleaning and disinfection* (MC 0.19, IQR 0.06; Table 1; Additional file 1: Table S4). The experts ranked *pig mixing* as second (MC 0.15, IQR 0.06), and third was *feed, water supply and bedding* (MC 0.13, IQR 0.08; Table 1 and Fig. 2). For the *setting* SAL-OUT, the experts ranked *pig mixing* (MC 0.19, IQR 0.07), *purchase of pigs or semen* (MC 0.19, IQR 0.07) and *feed*, *water supply and bedding* (MC 0.19, IQR 0.08) equally at the first place (Table 1 and Fig. 2). *Cleaning and disinfection* (MC 0.13, IQR 0.07) was ranked at the second place, while *other animals on farm* (including wildlife, MC 0.12, IQR 0.07) ranked third (Table 1 and Fig. 2). Details for the categories listed are provided in Table 1, Fig. 2 and Additional file 1: Tables S4–S5.

**Table 1:** Rank order of median weights for each biosecurity category in the different *settings* of pathogen and system

Scenario/rank	HEV-IN	HEV-OUT	SAL-IN	SAL-OUT
1	CnD	Mixing	CnD	Feed, water and bed; Mixing; Purchase
2	Mixing	Purchase	Mixing	CnD
3	Feed, water and bed	CnD	Feed, water and bed; Purchase	Animals
4	Transport	Feed, water and bed; Transport	Transport	Transport
5	Purchase	Equipment	Animals; Equipment; Humans	Equipment; Humans
6	Equipment; Humans	Humans		
7	Animals	Animals		

CnD, Cleaning and disinfection; Mixing, Pig mixing; Feed; Water and bed, feed, water supply, and bedding; Transport, Transport to/from farm; Purchase, Purchase of pigs or semen; Equipment, Material and equipment; Humans, Farm workers and visitors; Animals, Other animal species on farm, including wildlife. HEV-IN, HEV in indoor systems; HEV-OUT, HEV in outdoor systems, SAL-IN, *Salmonella* spp*.* in indoor systems, and SAL-OUT, *Salmonella* spp*.* in outdoor systems. Rank 1: most important

When considering HEV-IN, the biosecurity category *cleaning and disinfection* average relative weight ranked highest (MC 0.25, IQR 0.08), followed by *mixing of pigs* at second rank (MC 0.2, IQR 0.11), and *feed, water supply and bedding* (MC 0.13, IQR 0.06) at third rank (Table 1 and Fig. 2). For HEV-OUT, on average the experts ranked *pig mixing* (MC 0.25, IQR 0.23) as the most important category, then ranked *pig or semen purchase* second (MC 0.16, IQR 0.08) and *cleaning and disinfection* third (MC 0.13, IQR 0.13; Table 1 and Fig. 2). All other categories are listed in Table 1, Fig. 2 and Additional file 1: Tables 6–7. In general, the categories *animals* (other than pigs and including wildlife), *equipment*, *humans* ranked lowest in all four *settings*, except for *animals* in SAL-OUT (Table 1 and Fig. 2).

### Biosecurity categories with high-relevance/high-agreement measures

Several measures (94/222, 42.3%) across all four *settings* were consistently (IQR 0–1) considered highly relevant (MM 4–5), while the proportion of measures consistently (IQR 0–1) deemed of low relevance (MM 0–3) was comparatively small (10/222, 4.5%). Additionally, measures with high disagreement between the respondents (IQR ≥ 2.5) were not common (21/222, 9.**5**%), but more frequently identified in HEV-IN and HEV-OUT compared to SAL-IN and SAL-OUT (Table 2). A total of 97 measures across the four *settings* (97/222, 43.7%) were included in the group *all ranks/intermediate agreement* with IQR > 1 and < 2.5 (Table 2). Of these measures, 72.2% had high relevance (MM 4–5; 70/97), while the remaining 27.8% scored low in relevance (MM ≤ 3; 27/97; Additional file 1: Tables 4–7).

**Table 2:** Classification of biosecurity measures based on the relevance and the level of agreement between the experts

	HEV-IN		HEV-OUT		SAL-IN		SAL-OUT		Total	
	n	%	n	%	n	%	n	%	n	%
High relevance ranking (MM 4–5) and high agreement (IQR 0–1)	18	31.6	16	29.6	34	59.7	26	48.1	94	42.3
Low relevance ranking (MM ≤ 3) and high agreement (IQR 0–1)	2	3.5	3	5.6	2	3.5	3	5.6	10	4.5
All ranks, low agreement (IQR ≥ 2.5)	7	12.3	10	18.5	2	3.5	2	3.7	21	9.5
All ranks/intermediate agreement (IQR > 1 and < 2.5)	30	52.6	25	46.3	19	33.3	23	42.6	97	43.7
Total assessed measures	57	100	54	100	57	100	54	100	222	100

HEV-IN indicates HEV in indoor systems, HEV-OUT indicates HEV in outdoor systems, SAL-IN indicates *Salmonella* spp. in indoor systems, and SAL-OUT indicates *Salmonella* spp. in outdoor systems. MM indicates the biosecurity measure median relevance and IQR indicates the interquartile range

In the *setting* SAL-IN, a total of 34/57 measures (59.6%) were consistently ranked highly relevant (MM 4–5 and IQR 0–1). The categories *cleaning and disinfection*, *other animals on farm* (including wildlife)*, pig mixing, pig or semen purchase,* and *farm workers and visitors* had a greater number of high-relevance/high-agreement measures selected by the experts. In SAL-OUT, a total of 26/54 measures (48.1%) were consistently ranked as highly relevant, and mainly fell within the categories *purchase of pigs or semen*, and *pig mixing*. Concerning HEV-IN, a total of 18/57 consistently highly relevant (31.6%) measures were selected. *Cleaning and disinfection* and *pig mixing* were the two biosecurity categories with more measures selected by the experts. For HEV-OUT, 16/54 measures (29.6%) were consistently ranked as highly relevant, and *pig mixing* was the most included category. For each setting, high-relevance/high-agreement measures for the biosecurity categories selected in the first three ranks are listed in Table 3. The measures for all other biosecurity categories are listed in Additional file 1: Tables S4–S7.

**Table 3:** High-relevance/high-agreement measures listed for the biosecurity categories by *setting*, as determined by summaries of the relevance scores provided by experts

Biosecurity category	Specific biosecurity measure	HEV-IN	HEV-OUT	SAL-IN	SAL-OUT
CnD	The anteroom or hygiene lock and its equipment are cleaned and disinfected at least every 2 weeks			X	
	The feed storage and pipelines are cleaned and disinfected at least once a year			X	
	Dung from the sows held in farrowing pens is removed daily			X	
	The floor in each barn section and anteroom/hygiene locks (indoor) or in the changing room (outdoor) is even and without damage and thereby easy to clean and to disinfect. Also in outdoor systems, the huts are easy to clean and to disinfect			X	X
	Fields have a downtime and are not used for other livestock animals between pig holding				X
	The pit below slatted flooring is emptied between two batches	X			
	Corridors within barns are cleaned and disinfected before other pigs are moved via those corridors	X		X	
	The standard cleaning and disinfection procedures in the barns include drying	X		X	
	Sufficient downtime period after cleaning and disinfection before new pigs are moved into a cleaned and disinfected barn /compartment/ pen	X		X	
	Contact with manure is minimised by a suitable flooring system and cleaning application in the barns	X		X	
	Empty outdoor enclosure sections are given a sufficient downtime period before new pigs are moved onto it		X		X
Equipment	Machines/equipment are NOT shared with other farms or are cleaned and disinfected when returned	X	X	X	X
	Equipment (e.g., shovel, moving board) is NOT shared or it is cleaned and disinfected between age groups before use	X	X	X	X
	Dedicated injection syringes and needles are used for each age group and are cleaned and disinfected	X	X		
Feed, water and bedding	All feed and bedding are stored protected from wildlife, pets and pests	X	X	X	X
	Pigs have NO access to open water sources				X
	The drinking water is known to be free from or treated against microbiological contamination			X	X
Animals	The carcass storage is closed, so that wildlife, pets and pests DO NOT have access to the carcasses	X		X	X
	Rodent baits are used in the surroundings of the farm enclosures			X	
	All farm buildings are surrounded by a sufficient perimeter fence			X	
	Wild birds have NO access to the barn			X	
	Other livestock species on the farm are physically separated from the pigs			X	X
	Outdoor enclosures are surrounded by a sufficient perimeter fence				X
	A pest control program (against rodents, wild birds, insects) is carried out by a professional company			X	
	A pest control program (against rodents, wild birds, insects) is carried out			X	
Humans	All people have to wear farm-specific clothes and footwear		X	X	X
	Hygiene locks are present in sufficient number and at sensible locations (different age groups/ quarantine area) in the operation			X	
	Drivers of transport vehicles/distributors/other visitors have NO access to the barns (indoor) or outdoor enclosures (outdoor)			X	X
	Clothes and footwear are changed or cleaned and disinfected between outdoor enclosure sections or when moving to another production stage				X
	Changing rooms are present in sufficient number and at sensible locations (different age groups/ quarantine area) in the operation		X		
	All people have to use the hygiene lock when entering a barn	X		X	
Mixing	After weaning, weaners are NOT kept together with sows and piglets for some days/weeks, in the same farrowing room (indoor) or farrowing hut (outdoor)		X	X	X
	Stay-behinds and sick animals are isolated from the healthy ones	X	X	X	X
	Sick pigs are consistently handled after the healthy pigs	X	X	X	X
	Barn sections (indoor) or outdoor enclosures (outdoor) are managed all-in/all-out	X	X	X	X
	Cross-fostering is reduced to a minimum	X			
	Individual weaners or fatteners are NOT moved to another outdoor enclosure section, containing another group of animals of the same age		X		
	Cross-fostering does NOT occur four or more days after farrowing		X		X
Purchase	Purchased pigs have equal or better Salmonella / HEV status than own pigs	X	X	X	X
	The breeding pigs come from maximum one other farm of origin			X	X
	New pigs are quarantined for a sufficient period of time before entering the main herd			X	
	New breeding pigs are moved to a quarantine area for a sufficient period of time before they enter the main herd				X
	Purchased breeding pigs are moved to a quarantine area before they enter the herd	X		X	
	The fattening pigs come from maximum one other farm of origin		X		X
Transport	Transport vehicles (own or external) are cleaned and disinfected before loading pigs and do not already contain pigs of other farms	X	X	X	X
	External vehicles have NO access to the clean area within the farm perimeter			X	X
	A separate ramp or loading area is used so that pigs being loaded/unloaded do not come in contact with barns /compartments/ pens containing other pigs			X	X

CnD, Cleaning and disinfection; Mixing, Pig mixing; Feed, water and bed, Feed, water supply, and bedding; Transport, Transport to/from farm; Purchase, Purchase of pigs or semen; Equipment, Material and equipment; Humans, Farm workers and visitors; Animals, Other animal species on farm, including wildlife. HEV-IN, HEV in indoor systems; HEV-OUT, HEV in outdoor systems; SAL-IN, *Salmonella* spp. in indoor systems, and SAL-OUT, *Salmonella* spp. in outdoor systems

For the individual biosecurity measures there were similarities and differences between the four *settings*. Similarities were noticed across several *settings*: *pig mixing* was assessed as important in all four *settings,* although different measures were selected for each *setting* (Table 3). Furthermore, some measures in the *equipment* category were ranked as relevant, despite the category being ranked lower than other categories (Table 3). For *Salmonella* spp. several measures were commonly selected for both indoor and outdoor systems, and a high number of measures were listed as important overall (mainly in *cleaning and disinfection, other animals on farm* (including wildlife)*, and purchase of pigs or semen* categories), for HEV there was less consistency between the measures selected for indoor and outdoor systems, but more importance seemed again to be placed on *mixing of pigs* (Table 3). Furthermore, there were similarities between the two pathogens within one system: in indoor systems, *cleaning and disinfection* scored consistently high in relevance (Table 3). For outdoor systems, there were less biosecurity categories consistently being selected, and the measures flagged as relevant were mainly within the *pig mixing*, *cleaning and disinfection* and *purchase of pigs or semen* categories (Table 3).

### The experts’ comments and remarks

Some of the experts added comments on the biosecurity measures and the questionnaire. The comments broadly related to: the feasibility of implementing biosecurity measures, especially in outdoor *settings*; the lack of questions that explore interactions between the biosecurity measures (e.g. quarantine alongside testing of quarantined pigs); the lack of assessment of pig flow factors beyond mixing (e.g. status of incoming pigs and movements of pigs within a farm); missing elements on *Salmonella* spp. control in outdoor herds (e.g. avoiding use of lairages or minimising time in lairage, moving site as regularly as possible); the lack of greater detail in the definition of cleaning and disinfection methods (e.g., use of 'sweep-through' dung channel); and testing of workers specifically for *Salmonella* spp*.*

## Discussion

### Ranking of individual biosecurity categories and measures

The present study aimed to collect, weigh, and compare expert opinion on the relevance of biosecurity measures for the reduction of *Salmonella* spp*.* and HEV prevalence, in the indoor and outdoor European pig farming systems. The ranking and comparison of the biosecurity categories resulted in *pig mixing*; *cleaning and disinfection*; *feed, water and bedding*; and *purchase of pigs or semen* as the top four categories, while the four lowest ranked categories were *transport*, *equipment*, *animals* (other than pigs and including wildlife) and *humans*. Even though some categories were ranked higher than others, none stood out alone and all the biosecurity categories appeared to contribute at some level to the farm biosecurity, according to the experts. Similarities within husbandry system were identified, regardless of the pathogen: in indoor systems, the highest ranked biosecurity category was *cleaning and disinfection* followed by *pig mixing* and *feed, water supply and bedding*. In outdoor systems, *pig mixing* was consistently ranked higher. *Purchase of pigs or semen* were also considered to have great relevance, followed by *cleaning and disinfection*.

Within the biosecurity categories, several individual measures across all four *settings* were consistently considered highly relevant (high-ranked/high-agreement measures), and disagreement between respondents was not common, but more frequent in HEV-IN and HEV-OUT *settings* compared to SAL-IN and SAL-OUT. These findings can be partially explained by the lower number of expert responses for HEV that increased the uncertainty around the estimated relevance, but also it was likely to be driven by the lack of systematic research considering HEV in outdoor systems. The lack of high-ranked/high-agreement measures in some *settings* might in fact be due to the uncertainty that the experts have around the *settings* rather than the actual relevance of the measures. Furthermore, the country in which the experts operate and its epidemiological situation for *Salmonella* spp. and HEV, as much as the structure of farms and husbandry practices peculiar to the country might have determined different views. In a quantitative microbiological risk assessment undertaken in several European countries, some biosecurity practices (interventions at breeding farms or at abattoirs) were described to be most effective in high-seroprevalence countries while other practices (e.g. reduction of feed contamination) were more effective in low-seroprevalence countries [26]. Interestingly, measures which overlap in relevance between the two pathogens suggest that the implementation of these measures might be effective for both pathogens, according to respondents. To the authors’ knowledge, there is no active on-farm monitoring for HEV, nor are farmers forced to implement specific control measures, in contrast to monitoring and control programs for *Salmonella* spp. In case the transmission pathways have similar importance for both pathogens, there may be a collateral benefit when implementing biosecurity measures against *Salmonella* on circulation of HEV. Several highly ranked measures (MM 4–5) revealed a moderate variation in agreement (IQR > 1 and < 2.5) between the experts. The present work aimed at prioritising biosecurity measures with high relevance and high agreement as rated by most experts. As such high agreement was considered paramount in the selection of priority-measures. Measures with high relevance but low or intermediate agreement among experts may be effective in specific farming conditions and should still be considered for implementation on farm. The lower agreement may be indicative of the need for more research or research communication.

### Self-assessed knowledge and potential areas of study

The self-assessed knowledge score between the experts who answered one or more parts of the questionnaire varied by *setting*. The experts were more knowledgeable about SAL-IN, and the results suggest that the experts were less knowledgeable about HEV-OUT, which may reflect the experts own experience and/or the general knowledge available. While there is comparatively more literature available on the epidemiology of *Salmonella* spp. in pig farms and its main risk factors at different production levels (although there is little consensus on the most important factors) [14, 27–31]. HEV dynamics and predisposing factors have only been investigated more recently and there is less evidence on the effective interventions, as reviewed by Meester et al. [9]. Likewise, it is possible that outdoor systems are either less investigated in the scientific literature, or otherwise less represented in the countries selected for inclusion. Where this was not always the case, such as in the UK, the limited number of respondents might have diluted the contribution of those specific experts to the overall knowledge score. The smaller number of responses for HEV in outdoor systems added uncertainty to the EOE outputs, despite the fact that, overall, only moderately to very knowledgeable experts accessed the questions. Although knowledge was self-assessed and it could be overrated or underestimated [22], an assessment of the variation in responses by knowledge level was undertaken and there was no significant variation in the expert answers. As previously mentioned, the literature on the effectiveness of individual biosecurity measures is generally sparse for HEV and conflicting for *Salmonella* spp., thus these findings reinforce the importance of the expert opinion in narrowing down on measures that are effective.

### Pig mixing and purchase of pigs or semen

Findings from the EOE indicated that many of the individual measures related to limiting pig mixing at different rearing stages, as well as avoiding mixing of animals with different health status, were ranked as important for preventing *Salmonella* spp*.* and HEV spread, especially in outdoor systems. The limited amount of published findings supported some of these findings, with animals moved during the fattening period having increased odds of infection and pig mixing at the nursery stage and cross-fostering being a risk for HEV and *Salmonella* spp. occurrence [10, 30, 32, 33].

Purchasing semen, breeding or fattening pigs from a maximum of one farm of origin and with farms with equal or better *Salmonella* spp. status was regarded as highly important by the experts. Fewer related individual measures were ranked as highly important for HEV. Animal movement between farms and the introduction of infected pigs have been identified as risk factors on HEV prevalence on farm [34], and using a larger number of supplying herds (> 3) was a risk factor for *Salmonella* spp. [17]. Quarantining purchased pigs for a sufficient period before entering the main herd was identified as highly important in some *settings*, particularly for *Salmonella* spp.

### Cleaning and disinfection

*Cleaning and disinfection* was considered the most important biosecurity category for the prevention of *Salmonella* spp*.* and HEV in indoor systems (based on median category weight and interquartile range), with all the individual measures identified as highly important to at least one of the pathogens in indoor systems, whereas ensuring sufficient downtime between batches was highly important for all four *settings*. Regular, effective cleaning, disinfecting and drying of corridors, anterooms/hygiene locks, barns and barn applications were flagged as relevant measures for indoor systems, as well as having flooring systems and regimens that removed faeces away from pigs and were easy to clean. However, annual or more frequent cleaning and disinfection of feed storage and pipelines was only rated as highly important for *Salmonella* spp*.* in the indoor setting. The identification of all these factors may be related to the degree to which these have been studied previously, and these have been found to be important in a number of publications related to *Salmonella* spp*.* and HEV [35]. However, it should be noted that the exact details of the procedures and disinfectants used are not agreed upon in the literature and studies have shown variable or no effect, especially for *Salmonella* spp*.* control [36–38].

### Feed, water, and bedding, and other animal species on farm (including wildlife)

Protecting the stored feed and bedding from wildlife, pets and pests, was perceived as a highly important measure for the control of *Salmonella* spp*.* and HEV in both farming systems and stopping access of these to the carcase storage was considered highly important for all apart from the outdoor HEV setting. When it comes to reducing the prevalence of *Salmonella* spp*.,* especially in outdoor systems*,* water source and microbiological standards were rated as important. Water not sourced from the municipal supply has been associated with a 1.9 increase in the odds of infection, probably due to access to unclean waters [39]. The use of wet feed was consistently indicated as not very important; in the literature wet feed was found to either have a protective effect on *Salmonella* spp. infections or not, often also depending on form and grinding [14, 28, 40] and depending on whether the feed was acidified or not [41, 42]. The fact that in the EOE questionnaire it was not exactly stated whether the wet feed was fermented, and thus acidic, might have driven the low relevance score assigned by the experts.

A further four measures related to rodent and wild bird control were rated as highly important for *Salmonella* spp*.* in the indoor *setting*. These findings may reflect a consensus that control of wildlife is hard to achieve on outdoor farms, rather than the importance of these factors to controlling the pathogens. Studies have shown that the lack of rodent control is likely to increase the odds of being *Salmonella* spp*.* infected and that bird proof netting was a protective factor [39]. Ensuring physical separation from other livestock species and the presence of perimeter fences around enclosures was also rated highly for *Salmonella* spp*.* in both *settings*, which is supported by findings that using perimeter fences was a significant protective factor and allowing the contact of other animals with pigs increased the odds of *Salmonella* spp*.* seropositivity over four times [30].

### Material and equipment, farm workers and visitors and transport to/from farm

In general, *material and equipment, farm workers and visitors and transport to/from farm* categories were listed in the lower category ranks but some of the individual measures were consistently scored as highly relevant by the experts. For the control of both pathogens in indoor and outdoor systems, the experts agreed on the importance of not sharing farm machinery and equipment between farms, or, if that was the case, on the relevance of thorough cleaning and disinfection. Concerning HEV only, the experts flagged the use of dedicated injection syringes and needles for each age group, and their cleaning and disinfection. However, the importance of this route of HEV infection may be overstated as ineffective transmission of HEV through exposure to contaminated needles has been shown [43].

For both *Salmonella* spp. and HEV, cleaning and disinfection of transport vehicles before loading pigs and only collecting pigs from a single site were deemed important measures, but the effectiveness of these measures has not been clearly evidenced through previous research regarding *Salmonella* spp*.* [30]. When considering *Salmonella* spp*.* control, the experts placed high importance on using a separate loading area to load and unload pigs, and to avoid access of external vehicles or drivers to the farm clean area.

For farm workers and visitors, wearing farm-specific clothes and footwear was widely accepted as crucial to combat both *Salmonella* spp*.* and HEV (although not for HEV-Indoors). For *Salmonella* spp. in outdoor systems, changing or cleaning and disinfecting clothing and footwear between outdoor enclosure sections or when moving to another production stage was rated as highly important. Additionally, stopping visitors and transporter drivers accessing pig enclosures and barns was rated as important for *Salmonella* spp*.* in both *settings*. This was another area with scarce evidence from published studies, with some studies showing no effect or alternative effects [10] and although studies suggest that the strict observance of safety rules by farm workers and visitors could reduce transmission of pig viruses such as HEV, direct evidence is lacking [44]. Lastly, using hygiene locks and changing rooms for indoor farms was ranked high for both pathogens.

### Study limitations and validity

Although the experts were not selected through randomisation, the number and diversity of professional profiles was considered satisfactory and ranged from those that operate in the field and have hands-on experience of the pig farming industry across several European countries (48% of total respondents) to EBVS™ registered European Veterinary Specialists, being members of the European College of Veterinary Public Health (ECVPH) and or the European College of Porcine Health Management (ECPHM). The number of responses was also similar or greater than in other similarly structured studies [19, 20, 22, 25]. Despite the fact that EOE is a widely used technique, it is nonetheless important to consider the benefits and concern arising from the use of EOE, as much as the attainable standards with this specific technique [23, 45]. Additionally, the questionnaire design might have introduced some bias (e.g., questions for HEV and *Salmonella* spp*.* presented side-by-side), as the experts who scored a measure or category high for *Salmonella* spp*.* might have been more likely to score the measure or category also as high for HEV. Furthermore, it is likely that specific biosecurity measures, or groups of biosecurity measures, considered logical based on known transmission mechanisms might be overrepresented in the scientific research, potentially generating results biased towards certain measures. As the relevance of the biosecurity measures, as perceived by the experts, may represent the current knowledge on routes of transmission, future research or development of new farming concepts might shed more light on other or new transmission pathways, also changing the relative importance of on-farm biosecurity measures.

Although the respondents, especially field experts, may have unconsciously answered taking into account feasibility and costs of biosecurity measures, feasibility and costs were not explicitly evaluated. Further research that looks more precisely into this and that aims at involving other stakeholders (i.e. farmers, farm workers, traders) in the opinion analysis, might help in disentangling the underlying factors that play a role in the improvement of on-farm biosecurity [19, 46]. The synergies and associations between measures could not be accounted for, and no statistical tests to compare category weights or single measure relevance was undertaken. The application of statistical tests would imply the generalization to the opinion of a theoretical population of experts. However, we did not define such a homogenous population and did not try to obtain a representative sample of this expert population. Thus, we restricted our analyses to summarizing and describing the answers of the respondents to our EOE. These further steps could be taken forward in future studies, focusing on the synergies and associations between individual biosecurity measures, as much as on integrating information on the practical feasibility and economic impact of their implementation.

## Conclusions

This EOE demonstrated that experts identified the implementation of several measures from different biosecurity categories as important to prevent the introduction or limit the spread of *Salmonella* spp*.* and HEV infections on the farm. On farm activities related to cleaning and disinfection, as well as pig movement and mixing practices were perceived as mainly contributing to the protection of pigs against *Salmonella* spp*.* and HEV infection. The comparison of two different scenarios, indoor and outdoor systems, allowed for the identifications of differences in the biosecurity measures prioritised by the experts. In the literature, this is not often considered, and there is generally more research available on indoor farming. The uncertainty highlighted in the study around outdoor farming systems, especially in association with HEV, underlines the need for further research in such direction.

## Methods

An online questionnaire was created to evaluate and rank a list of selected biosecurity measures. The questionnaire was administered to European professionals who were asked to rank the biosecurity measures relevance in reducing the farm prevalence of the two pathogens.

### Expert selection

83 experts, from twelve European countries, were identified and invited to complete the questionnaire (Additional file 1: Table S1). Experts were recruited for having a high degree of expertise in the areas of pig farm biosecurity, *Salmonella* spp*.* and/or HEV presence/control on pig farms. It was aimed to identify experts with different professions comprising of researchers, veterinary practitioners and advisors, governmental staff and pig production consultants and experts.

### Questionnaire

Only biosecurity measures with published effectiveness in limiting or reducing *Salmonella* spp*.*/ HEV occurrence in the pig production chain were included in the questionnaire. An initial panel, including the authors and their institutional colleagues (eight persons assessing relevance for HEV, nine for *Salmonella* spp*.*), was used to select measures based on previous work undertaken, where published evidence was missing [47, 48]. Questions on different biosecurity measures were grouped in eight biosecurity categories: *pig mixing; cleaning and disinfection; feed and water supply, and bedding; transport to/from farm; purchase of pigs or semen; material and equipment; farm workers and visitors; other animal species on farm, including wildlife*. The questionnaire was created to evaluate the biosecurity measures in four different *settings*, defined as: 1) HEV in indoor systems (HEV-IN), 2) HEV in outdoor systems (HEV-OUT), 3) *Salmonella* spp*.* in indoor systems (SAL-IN), and 4) *Salmonella* spp*.* in outdoor systems (SAL-OUT). The invited experts were asked to answer questions for all *settings* but had the option to exclude those that were outside of their self-assessed expertise**.** Experts were requested to:

1. Rate their experience in the different farm systems, pathogens and for biosecurity in general by ranking their knowledge between 1 (not knowledgeable at all) and 5 (extremely knowledgeable).
2. Rank the effectiveness of the biosecurity categories. This was obtained by listing the above mentioned eight biosecurity categories and asking the experts to assign a score between 1 and 80 (accounting for the hypothetical scenarios of 10 equal points assigned to each of the eight categories) based on how important they considered them for the prevention/control of pathogen spread. A maximum of 80 points was allowed per *setting*.
3. Within each biosecurity category, score the relevance of each specific measure, according to its relevance for each *setting*, ranking each measure between 1 (least relevant) to 5 (most relevant).

At the end of the questionnaire, the experts were given the opportunity to provide ‘open text’ comments and remarks.

### Data collection

The questionnaire was designed in Qualtrics^XM®^ survey software, and definitions for specific terminology related to pig farming (e.g., barn section, loading area, pens, etc.) were added using pop-up boxes to provide clarity. Only the *settings* selected by the expert were used for the ranking of categories and relevance scoring of measures. The questionnaire was tested before release to ensure that it was easy to understand and to complete, run without errors and minor adjustments were made accordingly. The questionnaire was made available online, and a link was emailed to the invited experts along with a letter of invitation. The experts were requested to consent and had the option to stop responding at any point of the questionnaire. Responses were collected in the period between the 24th May 2021 and 30th July 2021.

### Data analysis

Responses were excluded from analysis if less than 70% of the questions of a selected *setting* were answered. All answers for a section of the questionnaire (pathogen or system) were excluded if the interviewee self-declared that their relevant level of expertise was “not knowledgeable at all”. Two approaches were applied to assess whether a possible association between the self-assessed level of expertise and the answers to the questions about biosecurity should be considered in further analyses. Multidimensional scaling (MDS) was used to reduce the answers to all the questions in a section into two dimensions. Answers were then plotted in a two-dimensional graph and labelled with the level of expertise of the respondent to the pathogen, the farming system or to biosecurity. Graphs were visually inspected to see if the clusters of answers corresponded to the categories of knowledge experts (statistical software, STATA 15). K-means cluster analyses with 2 and up to 5 clusters were applied to the answers per section. The proportions within the resulting clusters of knowledge levels of the respondents to the pathogen, the farming system and to biosecurity were compared to assess whether clusters of experts answering similarly would differ in their level of expertise (R version 4.0.3). Multidimensional scaling and graphical representation was performed using STATA 15 and K-means cluster analyses was performed with R version 4.0.3.

For analysis of the effectiveness of the biosecurity categories, a relative weight (RW) of importance for each category of biosecurity measures was calculated, scaled from 0 to 1 proportionally to the originally assigned score (between 1 and 80 points) for ease of analysis and comparison. Biosecurity measures were ordered within *settings* by (1) the median category weight (MC, largest to smallest) to highlight the category relevance, and (2) the category weight interquartile range (IQR, smallest to largest) to address the respondent variance. Additionally, within each biosecurity category the individual biosecurity measures were ranked by (1) the median measure relevance (MM, largest to smallest) to highlight the measure relevance, and (2) the median measure relevance interquartile range (IQR, smallest to largest) to address the respondent variance. Measures with MM above 4 were considered of high relevance, while measures with MM equal to or below 3 were considered of low relevance. Agreement between the experts was assessed using the IQR, where IQR 0–1 was considered a high level of agreement, and IQR equal to or higher than 2.5 was considered a low level of agreement. The measures were further classified in four categories by the relevance and agreement scores: (a) *high-ranked*/*high-agreement* measures, with MM 4–5 and IQR 0–1; (b) *low-ranked/high-agreement* measures, with MM ≤ 3 and IQR 0–1; (c) *all ranks/low agreement* measures, with MM 1–5 and IQR ≥ 2.5; (d) *all ranks/intermediate agreement* measures, with MM 1–5, IQR > 1 and < 2.5. These cut-offs were arbitrarily chosen by looking at the data distribution and introduced to simplify the data discussion and interpretation. These descriptive analyses were conducted in R version 4.0.3 [49].

## Supplementary Information

**Additional file 1**. **Table S1.** Number of invited experts and number of respondents kept in the analysis, by country. **Table S2.** Summary of the frequency of setting combination as selected in the 46 responses from experts that were kept in the analysis. **Table S3.** Participants’ self-assessed knowledge ranking in each of the five domains: on indoor farming systems, outdoor farming systems, *Salmonella* spp., HEV and on-farm biosecurity. **Tables S4-S7**. All biosecurity categories and all individual biosecurity measures included in the questionnaire and applied to the control of Salmonella spp. and HEV in indoor and outdoor farming, ranked by relevance and agreement. **Figure S1.** Distribution of all participants to the survey in the self-assessed knowledge ranks in percentages.

## Data Availability

Data can be made available upon reasonable request to the corresponding author.
