# Supplementary material for: Prioritization of pig farm biosecurity for control of Salmonella and hepatitis E virus infections: results of a European expert opinion elicitation
Source: Porcine Health Manag. 2023 Mar 6;9:8. doi: 10.1186/s40813-023-00306-0 (PMC9987137; doi:10.1186/s40813-023-00306-0)
Supplement: Supplementary file 1 — Additional file 1. Table S1. Number of invited experts and number of respondents kept in the analysis, by country. Table S2. Summary of the frequency of setting combination as selected in the 46 responses from experts that were kept in the analysis. Table S3. Participants’ self-assessed knowledge ranking in each of the five domains: on indoor farming systems, outdoor farming systems, Salmonella spp., HEV and on-farm biosecurity. Tables S4-S7. All biosecurity categories and all individual biosecurity measures included in the questionnaire and applied to the control of Salmonella spp. and HEV in indoor and outdoor farming, ranked by relevance and agreement. Figure S1. Distribution of all participants to the survey in the self-assessed knowledge ranks in percentages. [file 40813_2023_306_MOESM1_ESM.docx]

# Additional File 1

**Table 1.** Number of invited experts and number of respondents kept in the analysis (n, %), by country.

| **Country** | **Experts invited (n)** | **Responses analysed (n)** | **Percentage of total responses analysed (%)** |
| --- | --- | --- | --- |
| Germany | 16 | 11 | 23.9 |
| Poland | 9 | 7 | 15.2 |
| United Kingdom | 13 | 6 | 13.0 |
| Austria | 7 | 5 | 10.9 |
| Italy | 7 | 5 | 10.9 |
| Sweden | 8 | 4 | 8.7 |
| The Netherlands | 11 | 4 | 8.7 |
| Belgium | 3 | 1 | 2.2 |
| Bulgaria | 5 | 1 | 2.2 |
| Estonia | 1 | 1 | 2.2 |
| Norway | 1 | 1 | 2.2 |
| Czech Republic | 2 | 0 | 0.0 |
| **Total** | **83** | **46** | **100.0** |

**Table 2.** Summary of the frequency of *setting* combination as selected in the 46 responses from experts that were kept in the analysis.

| **Indoor systems** | **Outdoor systems** | ***Salmonella* spp*.*** | **HEV** | **n** | **%** |
| --- | --- | --- | --- | --- | --- |
| X | X | X |  | 13 | 28.3 |
| X |  | X |  | 10 | 21.7 |
| X |  | X | X | 9 | 19.6 |
| X | X | X | X | 7 | 15.2 |
| X |  |  | X | 4 | 8.7 |
| X | X |  | X | 3 | 6.5 |
|  |  |  |  | **Total 46** | **100.0%** |

Legend: the symbol ‘X’ indicates the settings selected by the experts.

**Table 3.** Participants’ self-assessed knowledge ranking in each of the five domains: on indoor farming systems, outdoor farming systems, *Salmonella* spp*.,* HEV and on-farm biosecurity.

|  | **Indoor system** | | **Outdoor system** | | ***Salmonella* spp*.*** | | **HEV** | | **Biosecurity** | |
| --- | --- | --- | --- | --- | --- | --- | --- | --- | --- | --- |
|  | n | % | n | % | n | % | n | % | n | % |
| Not knowledgeable at all | 0 | 0 | 5 | 10.9 | 2 | 4.3 | 10 | 21.7 | 0 | 0 |
| Slightly knowledgeable | 3 | 6.5 | 15 | 32.6 | 2 | 4.3 | 11 | 23.9 | 1 | 2.2 |
| Moderately knowledgeable | 10 | 21.7 | 18 | 39.1 | 14 | 30.4 | 16 | 34.8 | 10 | 21.7 |
| Very knowledgeable | 25 | 54.3 | 7 | 15.2 | 20 | 43.5 | 8 | 17.4 | 22 | 47.8 |
| Extremely knowledgeable | 8 | 17.4 | 1 | 2.2 | 8 | 17.4 | 1 | 2.2 | 13 | 28.3 |
| Total | 46 | 100 | 46 | 100 | 46 | 100 | 46 | 100 | 46 | 100 |

**Table 4**. Biosecurity categories and individual biosecurity measures applied to the control of *Salmonella* spp*.* in indoor farming, ranked by relevance and agreement.

| **Category** | **MC** | **IQR** | **Biosecurity measure** | **MM** | **IQR** | **n** |
| --- | --- | --- | --- | --- | --- | --- |
| CnD | 0.19 | 0.06 | **Dung from the sows held in farrowing pens is removed daily.** | **5** | **1** | **38** |
|  |  |  | **Corridors within barns are cleaned and disinfected before other pigs are moved via those corridors.** | **5** | **1** | **39** |
|  |  |  | **The floor in each barn section and anteroom or hygiene locks is even and without damage and thereby easy to clean and to disinfect.** | **5** | **1** | **39** |
|  |  |  | **The standard cleaning and disinfection procedures in the barns include dry.** | **5** | **1** | **38** |
|  |  |  | **Sufficient downtime period after cleaning and disinfection before new pigs are moved into a cleaned and disinfected barn /compartment/ pen.** | **5** | **1** | **38** |
|  |  |  | **Contact with manure is minimised by a suitable flooring system and cleaning application in the barns.** | **5** | **1** | **39** |
|  |  |  | **The anteroom or hygiene lock and its equipment are cleaned and disinfected at least every 2 weeks.** | **4** | **1** | **38** |
|  |  |  | **The feed storage and pipelines are cleaned and disinfected at least once a year.** | **4** | **1** | **39** |
|  |  |  | The pit below slatted flooring is emptied between two batches. | 4 | 2 | 37 |
|  |  |  | The drinking water system of the farm is cleaned and disinfected at least once a year. | 4 | 2 | 39 |
| Mixing | 0.15 | 0.06 | **Barn sections are managed all-in/all-out.** | **5** | **0** | **39** |
|  |  |  | **Sick pigs are consistently handled after the healthy pigs.** | **5** | **0.5** | **39** |
|  |  |  | **Stay-behinds and sick animals are isolated from the healthy ones.** | **5** | **1** | **38** |
|  |  |  | **After weaning, weaners are NOT kept together with sows and piglets for some days/ weeks, in the same farrowing room.** | **4** | **1** | **35** |
|  |  |  | Cross-fostering is reduced to a minimum. | 4 | 2 | 39 |
|  |  |  | Individual piglets are NOT cross-fostered more than once. | 4 | 2 | 39 |
|  |  |  | Cross-fostering does NOT occur 4 or more days after farrowing. | 4 | 2 | 37 |
|  |  |  | Individual weaners or fatteners are NOT moved to another pen, containing another group of animals of the same age. | 4 | 2 | 37 |
| Feed, water, and bedding | 0.13 | 0.08 | **All feed and bedding are stored protected from wildlife, pets and pests.** | **5** | **0** | **39** |
|  |  |  | **The drinking water is known to be free from or treated against microbiological contamination.** | **5** | **1** | **39** |
|  |  |  | The feed is coarsely ground (>1mm particle size). | 4 | 1.75 | 34 |
|  |  |  | All feed is tested for or treated against microbiological contamination. | 4 | 2 | 38 |
|  |  |  | The feed is acidified. | 4 | 2 | 38 |
|  |  |  | Wet feed is offered. | 3 | 1 | 34 |
| Purchase | 0.13 | 0.17 | **The breeding pigs come from maximum 1 other farm of origin.** | **5** | **1** | **38** |
|  |  |  | **Purchased pigs have equal or better Salmonella / HEV status than own pigs.** | **5** | **1** | **36** |
|  |  |  | **Purchased breeding pigs are moved to a quarantine area before they enter the herd.** | **5** | **1** | **39** |
|  |  |  | **New pigs are quarantined for a sufficient period of time before entering the main herd.** | **5** | **1** | **39** |
|  |  |  | The fattening pigs come from maximum 1 other farm of origin. | 4 | 2 | 38 |
|  |  |  | Semen originates from sources with equal or better Salmonella/ HEV status. | 3 | 4 | 35 |
| Transport | 0.10 | 0.06 | **External vehicles have NO access to the clean area within the farm perimeter.** | **5** | **1** | **39** |
|  |  |  | **A separate ramp or loading area is used so that pigs being loaded/unloaded do not come in contact with barns /compartments/ pens containing other pigs.** | **5** | **1** | **39** |
|  |  |  | **Transport vehicles (own or external) are cleaned and disinfected before loading pigs and do not already contain pigs of other farms.** | **5** | **1** | **39** |
|  |  |  | Separate transport vehicles are used for pigs of different age groups. | 4 | 2 | 38 |
| Equipment | 0.06 | 0.05 | **Machines/equipment are NOT shared with other farms or are cleaned and disinfected when returned.** | **5** | **0** | **39** |
|  |  |  | **Equipment (e.g., shovel, moving board) is NOT shared or it is cleaned and disinfected between age groups before use.** | **5** | **1** | **39** |
|  |  |  | Dedicated injection syringes and needles are used for each age group and are cleaned and disinfected. | 4 | 2 | 39 |
|  |  |  | Materials/tools (e.g., tail-docker, injection needles) are changed or cleaned and disinfected between litters of piglets. | 4 | 2 | 39 |
| Humans | 0.06 | 0.06 | **Hygiene locks are present in sufficient number and at sensible locations (different age groups/ quarantine area) in the operation.** | **5** | **1** | **39** |
|  |  |  | **All people have to use the hygiene lock when entering a barn.** | **5** | **1** | **39** |
|  |  |  | **All people have to wear farm-specific clothes and footwear.** | **5** | **1** | **39** |
|  |  |  | **Drivers of transport vehicles/distributors/other visitors have NO access to the barns.** | **5** | **1** | **39** |
|  |  |  | All people have to wash and disinfect their hands or wear disposable gloves before entering any barn. | 4 | 2 | 39 |
|  |  |  | Clothes and footwear are changed or cleaned and disinfected between barn sections or when moving to another production stage. | 4 | 2 | 39 |
|  |  |  | Disposable gloves are worn when manipulating carcasses and/or hands are washed and disinfected after manipulating carcasses. | 4 | 2 | 39 |
|  |  |  | All people have to wash and disinfect their hands or change disposable gloves between different barn section. | 4 | 2.5 | 39 |
|  |  |  | Adherence to a pig-contact free period before external people are allowed to enter the barn. | 3 | 2 | 39 |
|  |  |  | All people have to take a shower before entering any barn. | 2 | 1 | 39 |
| Animals | 0.06 | 0.08 | **The carcass storage is closed, so that wildlife, pets and pests DO NOT have access to the carcasses.** | **5** | **1** | **39** |
|  |  |  | **A pest control program (against rodents, wild birds, insects) is carried out.** | **5** | **1** | **39** |
|  |  |  | **Wild birds have NO access to the barn.** | **5** | **1** | **39** |
|  |  |  | **Other livestock species on the farm are physically separated from the pigs.** | **5** | **1** | **38** |
|  |  |  | **All farm buildings are surrounded by a sufficient perimeter fence.** | **4** | **1** | **39** |
|  |  |  | **A pest control program (against rodents, wild birds, insects) is carried out by a professional company.** | **4** | **1** | **37** |
|  |  |  | **Rodent baits are used in the surroundings of the farm enclosures.** | **4** | **1** | **36** |
|  |  |  | Cats and dogs have NO access to the barn. | 4 | 1.75 | 38 |
|  |  |  | NO other livestock species are present on the farm. | 3 | 2 | 39 |

Legend: CnD = cleaning and disinfection; Mixing = pig mixing, Feed, water and bed = feed, water supply, and bedding; Transport = transport to/from farm; Purchase = purchase of pigs or semen; Equipment = material and equipment; Humans = farm workers and visitors; Animals = other animal species on farm, including wildlife. The biosecurity categories are ranked by 1) relevance, indicated with the median category weight (MC, largest to smallest) and 2) between-participant agreement, indicated with the category weight interquartile range (IQR, smallest to largest). Individual biosecurity measures in each category are ranked by 1) relevance, indicated with the median relevance (MM, largest to smallest) and 2) between-participant agreement, indicated with the relevance interquartile range (IQR, smallest to largest). High-relevance/high-agreement measures are in bold letters.

**Table 5**. Biosecurity categories and individual biosecurity measures applied to the control of *Salmonella* spp*.* in outdoor farming, ranked by relevance and agreement.

| **Category** | **MC** | **IQR** | **Biosecurity measure** | **MM** | **IQR** | **n** |
| --- | --- | --- | --- | --- | --- | --- |
| Purchase | 0.19 | 0.07 | **The breeding pigs come from maximum 1 other farm of origin.** | **5** | **1** | **19** |
|  |  |  | **Purchased pigs have equal or better Salmonella / HEV status than own pigs.** | **5** | **1** | **18** |
|  |  |  | **New breeding pigs are moved to a quarantine area for a sufficient period of time before they enter the main herd.** | **5** | **1** | **20** |
|  |  |  | **The fattening pigs come from maximum 1 other farm of origin.** | **4** | **1** | **19** |
|  |  |  | Semen originates from sources with equal or better Salmonella / HEV status. | 1 | 1.5 | 15 |
| Mixing | 0.19 | 0.07 | **Outdoor enclosure sections are managed all-in/all-out.** | **5** | **0** | **20** |
|  |  |  | **Stay-behinds and sick animals are isolated from the healthy ones.** | **5** | **1** | **20** |
|  |  |  | **Sick pigs are consistently handled after the healthy pigs.** | **5** | **1** | **20** |
|  |  |  | **Cross-fostering does NOT occur 4 or more days after farrowing.** | **4** | **0.5** | **16** |
|  |  |  | **Weaners are NOT kept adjacent to farrowing sows and piglets in the farrowing hut, for some days/weeks after weaning.** | **4** | **1** | **18** |
|  |  |  | Individual weaners or fatteners are NOT moved to another outdoor enclosure section containing another group of animals of the same age. | 4 | 1.25 | 20 |
|  |  |  | Individual piglets are NOT cross-fostered more than once. | 4 | 2 | 20 |
|  |  |  | Cross-fostering is reduced to a minimum. | 4 | 2.25 | 20 |
| Feed, water, and bedding | 0.19 | 0.08 | **All feed and bedding are stored protected from wildlife, pets and pests.** | **5** | **0.25** | **20** |
|  |  |  | **Pigs have NO access to open water sources.** | **4** | **1** | **19** |
|  |  |  | **The drinking water is known to be free from or treated against microbiological contamination.** | **4** | **1** | **20** |
|  |  |  | All feed is tested for or treated against microbiological contamination. | 4 | 2 | 19 |
|  |  |  | The feed is coarsely ground (>1mm particle size). | 4 | 2 | 17 |
|  |  |  | The feed is acidified. | 4 | 2 | 19 |
|  |  |  | Wet feed is offered. | 3 | 1 | 18 |
| CnD | 0.13 | 0.07 | **Fields have a downtime and are not used for other livestock animals between pig holding.** | **5** | **0** | **17** |
|  |  |  | **Empty outdoor enclosure sections are given a sufficient downtime period before new pigs are moved onto it.** | **5** | **0.75** | **18** |
|  |  |  | **The floor in the changing room is even and without damages and also the huts are easy to clean and to disinfect.** | **4** | **1** | **19** |
|  |  |  | The standard cleaning and disinfection procedures for the outdoor equipment include dry. | 4 | 1.75 | 18 |
|  |  |  | The changing room and its equipment are cleaned and disinfected at least every 2 weeks. | 4 | 2 | 18 |
|  |  |  | The feed storage is cleaned and disinfected at least once a year. | 4 | 2.5 | 18 |
|  |  |  | The drinking water system of the farm is cleaned and disinfected at least once a year. | 4 | 3 | 17 |
|  |  |  | Dung from the sows held in farrowing huts is removed daily. | 3 | 2 | 18 |
|  |  |  | Contact with manure is minimised by a suitable flooring system and cleaning application in the hut. | 3 | 2 | 17 |
| Animals | 0.12 | 0.07 | **Outdoor enclosures are surrounded by a sufficient perimeter fence.** | **5** | **0** | **20** |
|  |  |  | **The carcass storage is closed, so that wildlife, pets and pests DO NOT have access to the carcasses.** | **5** | **1** | **20** |
|  |  |  | **Other livestock species on the farm are physically separated from the pigs.** | **5** | **1** | **20** |
|  |  |  | Rodent baits are used in the surroundings of the farm enclosures. | 5 | 1.25 | 16 |
|  |  |  | A pest control program (against rodents, wild birds, insects) is carried out. | 5 | 1.5 | 19 |
|  |  |  | A pest control program (against rodents, wild birds, insects) is carried out by a professional company. | 4 | 2 | 17 |
|  |  |  | Dogs have NO access to the outdoor enclosures. | 4 | 2 | 19 |
|  |  |  | NO other livestock species are present on the farm. | 2 | 1.25 | 20 |
| Transport | 0.11 | 0.06 | **External vehicles have NO access to the clean area within the farm perimeter.** | **5** | **1** | **20** |
|  |  |  | **A separate ramp or loading area is used so that pigs being loaded/unloaded do not come in contact with barns /compartments/ pens containing other pigs.** | **5** | **1** | **20** |
|  |  |  | **Transport vehicles (own or external) are cleaned and disinfected before loading pigs and do not already contain pigs of other farms.** | **5** | **1** | **20** |
|  |  |  | Separate transport vehicles are used for pigs of different age groups. | 3.5 | 1 | 20 |
| Equipment | 0.06 | 0.06 | **Machines/equipment are NOT shared with other farms or are cleaned and disinfected when returned.** | **5** | **0.25** | **20** |
|  |  |  | **Equipment (e.g., shovel, moving board) is NOT shared or it is cleaned and disinfected between age groups before use.** | **4.5** | **1** | **20** |
|  |  |  | Dedicated injection syringes and needles are used for each age group and are cleaned and disinfected. | 3.5 | 2.25 | 20 |
|  |  |  | Materials/tools (e.g., tail-docker, injection needles) are changed or cleaned and disinfected between litters of piglets. | 3.5 | 2.25 | 20 |
| Humans | 0.06 | 0.07 | **All people have to wear farm-specific clothes and footwear.** | **5** | **1** | **20** |
|  |  |  | **Drivers of transport vehicles/distributors/other visitors have NO access to the outdoor enclosures.** | **5** | **1** | **20** |
|  |  |  | Disposable gloves are worn when manipulating carcasses and/or hands are washed and disinfected after manipulating carcasses. | 5 | 1.25 | 20 |
|  |  |  | Changing rooms are present in sufficient number and at sensible locations (different age groups/ quarantine area) in the operation. | 4.5 | 2 | 20 |
|  |  |  | **Clothes and footwear are changed or cleaned and disinfected between outdoor enclosure sections or when moving to another production stage.** | **4** | **1** | **20** |
|  |  |  | All people have to wash and disinfect their hands or wear disposable gloves before entering any outdoor enclosure. | 4 | 1.5 | 20 |
|  |  |  | All people have to wash and disinfect their hands or change disposable gloves between different outdoor enclosure section. | 3 | 2 | 20 |
|  |  |  | Adherence to a pig-contact free period before external people are allowed to enter the outdoor enclosures. | 2 | 0.5 | 20 |
|  |  |  | All people have to take a shower before entering any outdoor enclosure. | 1 | 1 | 20 |

Legend: CnD = cleaning and disinfection; Mixing = pig mixing, Feed, water and bed = feed, water supply, and bedding; Transport = transport to/from farm; Purchase = purchase of pigs or semen; Equipment = material and equipment; Humans = farm workers and visitors; Animals = other animal species on farm, including wildlife. The biosecurity categories are ranked by 1) relevance, indicated with the median category weight (MC, largest to smallest) and 2) between-participant agreement, indicated with the category weight interquartile range (IQR, smallest to largest). Individual biosecurity measures in each category are ranked by 1) relevance, indicated with the median relevance (MM, largest to smallest) and 2) between-participant agreement, indicated with the relevance interquartile range (IQR, smallest to largest). High-relevance/high-agreement measures are in bold letters.

.

**Table 6**. Biosecurity categories and individual biosecurity measures applied to the control of HEV in indoor farming, ranked by relevance and agreement.

| **Category** | **MC** | **IQR** | **Biosecurity measure** | **MM** | **IQR** | **n** |
| --- | --- | --- | --- | --- | --- | --- |
| CnD | 0.25 | 0.08 | **The standard cleaning and disinfection procedures in the barns include dry.** | **5** | **1** | **22** |
|  |  |  | **Sufficient downtime period after cleaning and disinfection before new pigs are moved into a cleaned and disinfected barn /compartment/ pen.** | **5** | **1** | **22** |
|  |  |  | **Contact with manure is minimised by a suitable flooring system and cleaning application in the barns.** | **5** | **1** | **22** |
|  |  |  | The floor in each barn section and anteroom or hygiene locks is even and without damage and thereby easy to clean and to disinfect. | 5 | 1.5 | 23 |
|  |  |  | **Corridors within barns are cleaned and disinfected before other pigs are moved via those corridors.** | **4** | **1** | **22** |
|  |  |  | **The pit below slatted flooring is emptied between two batches.** | **4** | **1** | **22** |
|  |  |  | The anteroom or hygiene lock and its equipment are cleaned and disinfected at least every 2 weeks. | 4 | 2 | 22 |
|  |  |  | Dung from the sows held in farrowing pens is removed daily. | 4 | 2 | 22 |
|  |  |  | The feed storage and pipelines are cleaned and disinfected at least once a year. | 4 | 2 | 21 |
|  |  |  | The drinking water system of the farm is cleaned and disinfected at least once a year. | 4 | 3 | 21 |
| Mixing | 0.20 | 0.11 | **Barn sections are managed all-in/all-out.** | **5** | **0** | **23** |
|  |  |  | **Cross-fostering is reduced to a minimum.** | **5** | **1** | **22** |
|  |  |  | **Stay-behinds and sick animals are isolated from the healthy ones.** | **5** | **1** | **22** |
|  |  |  | **Sick pigs are consistently handled after the healthy pigs.** | **5** | **1** | **23** |
|  |  |  | After weaning, weaners are NOT kept together with sows and piglets for some days/ weeks, in the same farrowing room. | 5 | 2 | 20 |
|  |  |  | Individual piglets are NOT cross-fostered more than once. | 4 | 1.25 | 20 |
|  |  |  | Cross-fostering does NOT occur 4 or more days after farrowing. | 4 | 2 | 21 |
|  |  |  | Individual weaners or fatteners are NOT moved to another pen, containing another group of animals of the same age. | 4 | 2 | 23 |
| Feed, water, and bedding | 0.13 | 0.06 | **All feed and bedding are stored protected from wildlife, pets and pests.** | **5** | **1** | **22** |
|  |  |  | The drinking water is known to be free from or treated against microbiological contamination. | 4 | 2.5 | 23 |
|  |  |  | All feed is tested for or treated against microbiological contamination. | 2 | 2 | 23 |
|  |  |  | The feed is acidified. | 2 | 2 | 20 |
|  |  |  | The feed is coarsely ground (>1mm particle size). | 1.5 | 1 | 18 |
|  |  |  | Wet feed is offered. | 1 | 1 | 20 |
| Transport | 0.10 | 0.06 | **Transport vehicles (own or external) are cleaned and disinfected before loading pigs and do not already contain pigs of other farms.** | **5** | **1** | **23** |
|  |  |  | A separate ramp or loading area is used so that pigs being loaded/unloaded do not come in contact with barns /compartments/ pens containing other pigs. | 5 | 1.5 | 23 |
|  |  |  | External vehicles have NO access to the clean area within the farm perimeter. | 5 | 2 | 23 |
|  |  |  | Separate transport vehicles are used for pigs of different age groups. | 3 | 2 | 23 |
| Purchase | 0.09 | 0.12 | **Purchased pigs have equal or better Salmonella / HEV status than own pigs.** | **4** | **1** | **21** |
|  |  |  | **Purchased breeding pigs are moved to a quarantine area before they enter the herd.** | **4** | **1** | **21** |
|  |  |  | The fattening pigs come from maximum 1 other farm of origin. | 4 | 1.5 | 23 |
|  |  |  | The breeding pigs come from maximum 1 other farm of origin. | 4 | 1.75 | 22 |
|  |  |  | New pigs are quarantined for a sufficient period of time before entering the main herd. | 4 | 2 | 21 |
|  |  |  | Semen originates from sources with equal or better Salmonella/ HEV status. | 3 | 1.5 | 19 |
| Equipment | 0.06 | 0.06 | **Dedicated injection syringes and needles are used for each age group and are cleaned and disinfected.** | **5** | **0** | **22** |
|  |  |  | **Machines/equipment are NOT shared with other farms or are cleaned and disinfected when returned.** | **5** | **1** | **23** |
|  |  |  | **Equipment (e.g., shovel, moving board) is NOT shared or it is cleaned and disinfected between age groups before use.** | **5** | **1** | **22** |
|  |  |  | Materials/tools (e.g., tail-docker, injection needles) are changed or cleaned and disinfected between litters of piglets. | 5 | 2 | 23 |
| Humans | 0.06 | 0.07 | All people have to wear farm-specific clothes and footwear. | 5 | 1.5 | 23 |
|  |  |  | **All people have to use the hygiene lock when entering a barn.** | **4.5** | **1** | **22** |
|  |  |  | Drivers of transport vehicles/distributors/other visitors have NO access to the barns. | 4 | 1.5 | 23 |
|  |  |  | Hygiene locks are present in sufficient number and at sensible locations (different age groups/ quarantine area) in the operation. | 4 | 2 | 22 |
|  |  |  | Clothes and footwear are changed or cleaned and disinfected between barn sections or when moving to another production stage. | 4 | 2 | 22 |
|  |  |  | Disposable gloves are worn when manipulating carcasses and/or hands are washed and disinfected after manipulating carcasses. | 4 | 2 | 23 |
|  |  |  | All people have to wash and disinfect their hands or wear disposable gloves before entering any barn. | 3 | 2 | 23 |
|  |  |  | Adherence to a pig-contact free period before external people are allowed to enter the barn. | 3 | 2 | 23 |
|  |  |  | All people have to wash and disinfect their hands or change disposable gloves between different barn section. | 3 | 3 | 22 |
|  |  |  | All people have to take a shower before entering any barn. | 2 | 2 | 22 |
| Animals | 0.05 | 0.06 | **The carcass storage is closed, so that wildlife, pets and pests DO NOT have access to the carcasses.** | **5** | **1** | **22** |
|  |  |  | A pest control program (against rodents, wild birds, insects) is carried out. | 4 | 2 | 23 |
|  |  |  | Rodent baits are used in the surroundings of the farm enclosures. | 4 | 2 | 21 |
|  |  |  | Other livestock species on the farm are physically separated from the pigs. | 4 | 2 | 23 |
|  |  |  | All farm buildings are surrounded by a sufficient perimeter fence. | 4 | 2.5 | 23 |
|  |  |  | Cats and dogs have NO access to the barn. | 3.5 | 3 | 22 |
|  |  |  | A pest control program (against rodents, wild birds, insects) is carried out by a professional company. | 3 | 1.5 | 23 |
|  |  |  | Wild birds have NO access to the barn. | 3 | 2.75 | 22 |
|  |  |  | NO other livestock species are present on the farm. | 3 | 3.5 | 22 |

Legend: CnD = cleaning and disinfection; Mixing = pig mixing, Feed, water and bed = feed, water supply, and bedding; Transport = transport to/from farm; Purchase = purchase of pigs or semen; Equipment = material and equipment; Humans = farm workers and visitors; Animals = other animal species on farm, including wildlife. The biosecurity categories are ranked by 1) relevance, indicated with the median category weight (MC, largest to smallest) and 2) between-participant agreement, indicated with the category weight interquartile range (IQR, smallest to largest). Individual biosecurity measures in each category are ranked by 1) relevance, indicated with the median relevance (MM, largest to smallest) and 2) between-participant agreement, indicated with the relevance interquartile range (IQR, smallest to largest). High-relevance/high-agreement measures are in bold letters.

**Table 7**. Biosecurity categories and individual biosecurity measures applied to the control of HEV in outdoor farming, ranked by relevance and agreement.

| **Category** | **MC** | **IQR** | **Biosecurity measure** | **MM** | **IQR** | **n** |
| --- | --- | --- | --- | --- | --- | --- |
| Mixing | 0.25 | 0.23 | **Outdoor enclosure sections are managed all-in/all-out.** | **5** | **0** | **10** |
|  |  |  | **Weaners are NOT kept adjacent to farrowing sows and piglets in the farrowing hut, for some days/weeks after weaning.** | **5** | **1** | **9** |
|  |  |  | **Individual weaners or fatteners are NOT moved to another outdoor enclosure section, containing another group of animals of the same age.** | **5** | **1** | **10** |
|  |  |  | **Stay-behinds and sick animals are isolated from the healthy ones.** | **5** | **1** | **10** |
|  |  |  | **Sick pigs are consistently handled after the healthy pigs.** | **4.5** | **1** | **10** |
|  |  |  | **Cross-fostering does NOT occur 4 or more days after farrowing.** | **4** | **1** | **9** |
|  |  |  | Cross-fostering is reduced to a minimum. | 4 | 3 | 9 |
|  |  |  | Individual piglets are NOT cross-fostered more than once. | 3.5 | 1.25 | 8 |
| Purchase | 0.16 | 0.08 | New breeding pigs are moved to a quarantine area for a sufficient period of time before they enter the main herd. | 4.5 | 1.75 | 10 |
|  |  |  | **The fattening pigs come from maximum 1 other farm of origin.** | **4** | **1** | **10** |
|  |  |  | **Purchased pigs have equal or better Salmonella / HEV status than own pigs.** | **4** | **1** | **9** |
|  |  |  | The breeding pigs come from maximum 1 other farm of origin. | 4 | 1.75 | 10 |
|  |  |  | Semen originates from sources with equal or better Salmonella / HEV status. | 1.5 | 2 | 8 |
| CnD | 0.13 | 0.13 | **Empty outdoor enclosure sections are given a sufficient downtime period before new pigs are moved onto it.** | **5** | **1** | **10** |
|  |  |  | Fields have a downtime and are not used for other livestock animals between pig holding. | 5 | 1.75 | 10 |
|  |  |  | The standard cleaning and disinfection procedures for the outdoor equipment include dry. | 4.5 | 1.25 | 8 |
|  |  |  | The changing room and its equipment are cleaned and disinfected at least every 2 weeks. | 4 | 1.75 | 10 |
|  |  |  | The floor in the changing room is even and without damages and also the huts are easy to clean and to disinfect. | 4 | 1.75 | 10 |
|  |  |  | Contact with manure is minimised by a suitable flooring system and cleaning application in the hut. | 4 | 2 | 9 |
|  |  |  | The feed storage is cleaned and disinfected at least once a year. | 4 | 2 | 9 |
|  |  |  | The drinking water system of the farm is cleaned and disinfected at least once a year. | 4 | 3 | 9 |
|  |  |  | Dung from the sows held in farrowing huts is removed daily. | 3.5 | 1.75 | 10 |
| Feed, water, and bedding | 0.12 | 0.11 | **All feed and bedding are stored protected from wildlife, pets and pests.** | **4.5** | **1** | **10** |
|  |  |  | Pigs have NO access to open water sources. | 4 | 1.75 | 10 |
|  |  |  | The drinking water is known to be free from or treated against microbiological contamination. | 4 | 2.5 | 10 |
|  |  |  | All feed is tested for or treated against microbiological contamination. | 2.5 | 2.5 | 10 |
|  |  |  | The feed is coarsely ground (>1mm particle size). | 1 | 0 | 10 |
|  |  |  | Wet feed is offered. | 1 | 0 | 10 |
|  |  |  | The feed is acidified. | 1 | 1 | 10 |
| Transport | 0.12 | 0.18 | **Transport vehicles (own or external) are cleaned and disinfected before loading pigs and do not already contain pigs of other farms.** | **5** | **1** | **10** |
|  |  |  | A separate ramp or loading area is used so that pigs being loaded/unloaded do not come in contact with barns /compartments/ pens containing other pigs. | 5 | 1.75 | 10 |
|  |  |  | External vehicles have NO access to the clean area within the farm perimeter. | 5 | 2 | 10 |
|  |  |  | Separate transport vehicles are used for pigs of different age groups. | 3 | 2.25 | 10 |
| Equipment | 0.06 | 0.06 | **Machines/equipment are NOT shared with other farms or are cleaned and disinfected when returned.** | **5** | **0.75** | **10** |
|  |  |  | **Equipment (e.g., shovel, moving board) is NOT shared or it is cleaned and disinfected between age groups before use.** | **5** | **0.75** | **10** |
|  |  |  | **Dedicated injection syringes and needles are used for each age group and are cleaned and disinfected.** | **5** | **0.75** | **10** |
|  |  |  | Materials/tools (e.g., tail-docker, injection needles) are changed or cleaned and disinfected between litters of piglets. | 4 | 2 | 10 |
| Humans | 0.04 | 0.04 | **All people have to wear farm-specific clothes and footwear.** | **5** | **1** | **10** |
|  |  |  | Disposable gloves are worn when manipulating carcasses and/or hands are washed and disinfected after manipulating carcasses. | 4.5 | 1.75 | 10 |
|  |  |  | **Changing rooms are present in sufficient number and at sensible locations (different age groups/ quarantine area) in the operation.** | **4** | **1** | **9** |
|  |  |  | Clothes and footwear are changed or cleaned and disinfected between outdoor enclosure sections or when moving to another production stage. | 4 | 1.5 | 10 |
|  |  |  | Drivers of transport vehicles/distributors/other visitors have NO access to the outdoor enclosures. | 4 | 3.5 | 10 |
|  |  |  | All people have to wash and disinfect their hands or wear disposable gloves before entering any outdoor enclosure. | 3.5 | 1 | 10 |
|  |  |  | All people have to wash and disinfect their hands or change disposable gloves between different outdoor enclosure section. | 3 | 1.5 | 10 |
|  |  |  | Adherence to a pig-contact free period before external people are allowed to enter the outdoor enclosures. | 2 | 1.75 | 10 |
|  |  |  | All people have to take a shower before entering any outdoor enclosure. | 1.5 | 1.75 | 10 |
| Animals | 0.03 | 0.05 | The carcass storage is closed, so that wildlife, pets and pests DO NOT have access to the carcasses. | 4.5 | 1.75 | 10 |
|  |  |  | Outdoor enclosures are surrounded by a sufficient perimeter fence. | 4.5 | 2 | 10 |
|  |  |  | A pest control program (against rodents, wild birds, insects) is carried out. | 4 | 2.5 | 10 |
|  |  |  | Rodent baits are used in the surroundings of the farm enclosures. | 3.5 | 2.5 | 10 |
|  |  |  | Other livestock species on the farm are physically separated from the pigs. | 3.5 | 2.75 | 10 |
|  |  |  | A pest control program (against rodents, wild birds, insects) is carried out by a professional company. | 3 | 1.75 | 10 |
|  |  |  | Dogs have NO access to the outdoor enclosures. | 3 | 3.5 | 10 |
|  |  |  | NO other livestock species are present on the farm. | 2 | 2.75 | 10 |

Legend: CnD = cleaning and disinfection; Mixing = pig mixing, Feed, water and bed = feed, water supply, and bedding; Transport = transport to/from farm; Purchase = purchase of pigs or semen; Equipment = material and equipment; Humans = farm workers and visitors; Animals = other animal species on farm, including wildlife. The biosecurity categories are ranked by 1) relevance, indicated with the median category weight (MC, largest to smallest) and 2) between-participant agreement, indicated with the category weight interquartile range (IQR, smallest to largest). Individual biosecurity measures in each category are ranked by 1) relevance, indicated with the median relevance (MM, largest to smallest) and 2) between-participant agreement, indicated with the relevance interquartile range (IQR, smallest to largest). High-relevance/high-agreement measures are in bold letters.

**Figure 1.** Distribution of all participants to the survey (n=46) in the self-assessed knowledge ranks in percentages. Legend: 1=not knowledgeable at all, 2=slightly knowledgeable, 3=moderately knowledgeable, 4=very knowledgeable, 5=extremely knowledgeable. Colour legend, cut-offs were defined based on the percent of total responses, at: 1-5%; 6-10%; 11-15%; 16-20%; 21-25%; 26-30%; 31-35%; 36-40% (from lighter to darker grey shade).
